# Drug abuse risk and protective factors among Hispanic adolescents^☆^

**DOI:** 10.1016/j.pmedr.2016.01.012

**Published:** 2016-02-03

**Authors:** Steven Schinke, Traci Schwinn, Jessica Hopkins, Lindsey Wahlstrom

**Affiliations:** a School of Social Work, Columbia University, New York, NY, United States; b Trial Reach, Inc., New York, NY, United States

**Keywords:** Hispanic adolescents, Drug use, Risk and protective factors

## Abstract

Drug use is a disquieting problem among the growing population of U.S. Hispanic adolescents. This study examined risk and protective factors associated with drug use in a sample of 507 Hispanic adolescents. Data were collected in 2014 and 2015 from youths across the United States. The sample had a mean age of 14.1 years and was 37.3% male. Youths responded to an online questionnaire about their drug use and risk and protective factors associated with drug use. Negative self-image, higher levels of stress, weaker coping skills, peer drug use, lower levels of self-control, goal-setting, problem-solving skills, and self-efficacy, and higher intentions to use drugs in the future were associated with increased odds of past-month drug use. Youths with higher self-images who spoke mostly English at home were less likely to use drugs than youths with higher self-images who spoke mostly Spanish at home.Study results have implications for gaining a better understanding of drug use risk and protective factors among America's growing population of Hispanic adolescents. Efforts to better examine and enhance Hispanic youths' cognitive-behavioral skills seem a wise investment.

## Introduction

Notwithstanding their increased numbers, U.S. Hispanic adolescents have not received commensurate levels of research attention on their health behavior risks. Among those risks is drug use. Relative to their non-Hispanic Black and White classmates, 9th-grade Hispanic youths have higher use rates of marijuana, alcohol, inhalants, cocaine, heroin, ecstasy, methamphetamines, hallucinogens, injected drugs, steroids, and prescription drugs taken for recreational purposes, as well as higher rates of binge drinking (Center for Disease Control and Prevention (CDC), 2014). Ever-smoking rates for cigarettes among 9th-grade Hispanic youths are also higher than rates for non-Hispanic Black and White youths (CDC, 2014).

Explanations for high rates of drug use among Hispanic adolescents include peer pressure, stress, developmental influences, and negative role models (Goldberg, 2013; Pokhrel, Herzog, Sun, Rohrbeck, & Sussman, 2013; Kopak, 2014, Zapata Roblyer et al., 2015). Also implicated are discrimination, acculturation, family influences, access to illicit substances, misperceptions about risks of drug use, and reduced social disapproval of drug use (De La Rosa et al., 2015, Kulis et al., 2009, Prado et al., 2008, Otiniano Verissimo et al., 2014). Less is known about cognitive and behavioral variables linked with drug use risk and protective factors among Hispanic youth.

These include variables identified in learning theory and social cognitive theory that may help explain youths' risk for drug use. Learning theory explains how people learn through observation, modeling, and rewards (Bandura, 1977). Social cognitive theory predicts behavior through mediating channels of self-efficacy, intentions, and related perceptions (Bandura, 1986, Bandura, 1997). Embedded in both theories are cognitive-behavioral variables associated with drug use. Together with knowledge of youths' demography and cultural orientations, data on these variables can shed light on risk and protective factors associated with drug use among Hispanic youth.

The purpose of the present study was to empirically investigate the relationship between known risk and protective factors and drug use behavior among Hispanic youth. In particular, based on our theoretical framework, we hypothesized that peer influences and a constellation of cognitive-behavioral risk and protective factors would reveal drug use associations. Reflecting prior work on the role of acculturative factors on substance use among Hispanic youth, we further hypothesized that youths' levels of acculturation would moderate their reported drug use. The objective of the study, therefore, was to examine the salience of theoretically identified cognitive-behavioral variables, together with demographic and cultural variables, on the prevalence of illicit substance use among a representative sample of Hispanic youth in the United States.

## Methods

### Design

The study was conducted in late 2014 and early 2015. As part of a larger clinical trial, baseline data were collected from a sample of Hispanic adolescents recruited from community-based and social services organizations across the U.S. Once youths assented to study participation and obtained parental permission, they received electronic links to secure online questionnaires. Upon submission of their completed questionnaires, youths earned small online gift certificates for retail merchants.

### Sample

The study sample was 507 Hispanic youths. Following recruitment, 518 Hispanic adolescents registered for the study; 11 did not complete the questionnaire. The average questionnaire completion time, from the time youths received the questionnaire until the time they submitted the questionnaire, was 1.6 days.

### Measurement

Study questionnaires covered risk and protective factors linked with substance use and identified in the theoretical and empirical literature. On the questionnaires, youths responded to questions about their Hispanic heritage and other acculturation parameters (α = .84, Phinney, 1992; α = .81, Wallen, Feldman, & Anliker, 2002; α = .79, Unger et al., 2002). Reported by monthly frequencies of use, drug use items on the questionnaires covered all commonly used substances (test–retest *r* = .82–.95, Centers for Disease Control and Prevention, 2004). Risk and protective factor items on the questionnaires were Likert-scored on 4-point scales. These items asked youths about their self-image (Blascovich & Tomaka, 1993), behavioral intentions (α = .77, Hansen, 1996), ability to set goals (α = .76, Fearnow-Kenney, Hansen, & Ralph, 2002), self-control (α **=** **.**83–.85, Tangney, Baumeister, & Boone, 2004), stress (α **=** **.**74–.86, Cohen, Kamarck, & Mermelstein, 1983), media resistance (α = .87 Primack, Gold, Switzer, Hobbs, Land, & Fine, 2006), problem solving (α = .82, Heppner & Peterson, 1982), coping skills (α **=** .81, Seiffge-Krenke, 1995), self-efficacy (α **=** **.**84, Macaulay, Griffin, & Botvin, 2002), and peer influences (α **=** **.**70–.91, Santor, Messervey, & Kusumakar, 2000).

### Data analysis

Risk and protective factor composite scores were computed by averaging responses to all items for each factor. Owing to expectedly low base rates of drug use—given youths' ages—reported use rates were aggregated across alcohol, cigarettes, marijuana, inhalants, ecstasy, mushrooms, cocaine, methamphetamines, prescription pills, and heroin. Logistic regression juxtaposed youths' drug use with their scores on acculturation measures and on the measured risk and protective factor variables. Yielding odds ratios and 95% confidence intervals, these analyses were computed in unadjusted models and in models adjusted for age, gender, race, living arrangement, parent education, and language spoken at home.

## Results

Demographic and drug use prevalence data for the sample appear in Table 1. Regarding youths' reported races, the large percentage of the “Other” racial classification resulted from youths choosing “Hispanic” as their ethnicity and race. To test our hypothesis about the influence of risk and protective factors, we examined the contribution of each measured cognitive-behavioral variable associated with youths' past-month drug use. According to the adjusted analytic model, youths with higher self-images were less likely to use drugs than youths with lower self-images (*OR* = 0.50, 95% *CI* = 0.25, 0.99, *p* < .05; Table 2). Youths with lower levels of stress were less likely to use drugs than youths with higher levels of stress (*OR* = 0.56, 95% *CI* = 0.38, 0.84, *p* < .01). Compared to youths with weaker coping skills, those with stronger coping skills were less likely to use drugs (*OR* = 0.28, 95% *CI* = 0.13, 0.57, *p* < .001). Youths whose peers used drugs, relative to youths whose peers did not use drugs, were more likely to use drugs (*OR* = 7.52, 95% *CI* = 2.80, 20.22, *p* < .001). Youths with higher levels of self-control were less likely to use drugs than youths with lower levels of self-control (*OR* = 0.60, 95% *CI* = 0.40, 0.91, *p* < .05). Relative to youths with lower goal setting skills, youths with higher goal setting skills were less likely to use drugs (*OR* = 0.47, 95% *CI* = 0.27, 0.81, *p* < .01). Youths with better problem-solving skills (*OR* = 0.40, 95% *CI* = 0.21, 0.77, *p* < .01) and greater self-efficacy (*OR* = 0.35, 95% *CI* = 0.16, 0.78, *p* < .01) were less likely to use drugs. Youths' intentions to use drugs in the future were associated with past month drug use (*OR* = 22.29, 95% *CI* = 9.82, 50.58, *p* < .001).

To test the moderating influence of acculturative factors, we examined the interaction of those factors with the measured cognitive-behavioral variables to explain youths' past-month drug use. One relationship was significant. Among youths who had positive self-images, those who primarily spoke Spanish at home were more likely to use drugs use than those who primarily spoke English at home (*OR* = 7.89, *CI* = 1.21, 51.56, *p* < .05).

## Discussion

Study data reveal associations between risk and protective factors and past-month drug use among a geographically diverse sample of Hispanic adolescents. Findings that youths' drug use reports were linked with cognitive and behavioral variables known to lower or to raise drug use risks among adolescents advances knowledge on the salience of these relationships for Hispanic youth. The rapid population growth of Hispanic youth in the United States, together with trends toward greater drug use among these U.S. Hispanic youth adds value to study data confirming factors associated with increased or decreased drug use by Hispanic adolescents. Interactions in the data for youths' self-images and for their speaking English or Spanish at home with youths' drug use further contribute knowledge on the epidemiology of drug use in this important population. Among the implications of these findings is that drug use research among U.S. Hispanic adolescents may gain precision by encompassing the entirety of cultural and psychosocial variables that surround these youths and that may influence their attitudes and behavior.

Study data mirror other findings that less drug use among Hispanic youth is associated with a positive self-image, lower levels of perceived stress, higher levels of skills related to coping with stress, self-control abilities, self-efficacy, goal setting, and problem solving (Gil et al., 2004, Zapata Roblyer et al., 2015). Also in line with other data are results on the degree to which peer influence is associated with Hispanic youths' alcohol, marijuana, tobacco, and other drug use (Bacio et al., 2013, Szapocznik and Williams, 2000).

That youths with higher self-images who spoke mostly English at home were less likely to use drugs than youths with higher self-images who spoke mostly Spanish at home is also reflected in prior work (Guilamo-Ramos, Jaccard, Johansson, & Turrisi, 2004). Warranting mention, however (and remaining untested in our study) is that this relationship may be strongest among Hispanic families who recently relocated to the U.S. and who speak more Spanish than English at home. For example, Guilamo-Ramos et al. (2004) posit that recently immigrated families may encounter greater levels of stress, thereby increasing the likelihood of youths' substance use. In line with acculturation stress theory, Guilamo-Ramos et al. suggest that Hispanic youths from families who are more established in the U.S. and who speak more English than Spanish at home may be more likely to use harmful substances.

By far, the largest associations uncovered by our analysis substantiated the relationship between youths' current drug use and their intentions to use drugs in the future, a finding reported by Skenderian, Siegel, Crano, Alvaro, and Lac (2008), among others. The strength of these data begs for further explanation. If intentions drive behavior, alterations in the former may impact the latter. If current drug use drives future intentions, youths may realize a self-fulfilling prophecy. Either way, drug use intentions may be construed as a multiplicity of cognitive processes—including youths' plans to use drugs, predictions based on what youths' age-mates are doing, self-images of one's own future behavior, and a fatalistic worldview.

Study data are particularly informative when viewed within the theoretical framework that guided selection of the measured risk and protective factors. Applied to drug use among youth, learning theory and cognitive–behavioral theory reveal the value of coping abilities to buffer the stressors of the adolescent years. Similarly, findings on the relationship between drug use and youths' self-control, goal setting, self-efficacy, and problem-solving skills fit well within a framework that explains risk and protective factors on the basis of learned cognitive–behavioral responses. Moreover, data from this study underscore the centrality of perceived and actual peer influences as contributors to drug use among Hispanic youth.

Neither our data nor any other comparable findings can definitively clarify the role of acculturation to explain drug use among Hispanic youth. By collecting and reporting salient new data on this topic, investigators will iteratively build a knowledge base as Hispanic–American youth grow in numbers and potentially encounter drug use.

Lending strength to our findings is the breadth of the sample. Coming from more than one-half of the states in the U.S., including states with the largest populations of Hispanic people, the sample is geographically diverse. Also strengthening the study was the measurement battery that covered drug use risk and protective factors not usually included in assessments with Hispanic youths. Nonetheless, the data are limited by the study's cross-sectional design and relatively small sample. Too, youths were recruited from third-party human services agencies, suggesting that study participants may have been already involved with structured programs and not at high risk for drug use. The self-report nature of youths' responses also limits our findings.

Notwithstanding its limitations, the study uncovered risk and protective factors that may help explain drug use among Hispanic youths. Perhaps our data will encourage focused research with Hispanic adolescents to disaggregate factors that contribute to drug use. Ultimately, studies of why some Hispanic adolescents use drugs and why others do not will stimulate efforts to help Hispanic youths avoid harmful drug use.

## Conflict of interest statement

The authors declare that there are no conflicts of interest in the conduct and reporting of this study.

## Figures and Tables

**Table 1:** Demographic and drug use prevalence data for a U.S. sample of Hispanic adolescents in 2014 and 2015 (*N* = 507).

Variable	%	*M*	*SD*
Age (years)		14.10	1.10
Female	62.7		
Race			
White	27.0		
Black	4.6		
Other	68.4		
Ethnicity			
Hispanic	100		
Living arrangement			
Mother and father	59.7		
Single parent or two-parent with a step-parent	36.7		
Grandparents, aunt and/or uncle, other	3.6		
Language Spoken at Home			
Spanish only or mostly Spanish	31.3		
Spanish and English equally	28.7		
English only or mostly English	40.0		
Parent education			
2 or less years of college	54.3		
2 or more years of college	23.6		
Unknown	22.1		
Geographic area			
Northeast	30.3		
West	29.9		
South	23.8		
Midwest	16.0		
Drug use, past 30 days			
Marijuana	4.1		
Alcohol	7.3		
Tobacco	1.4		
Inhalants, ecstasy, mushrooms, cocaine, methamphetamines, prescription pills, or heroin	3.0		

**Table 2:** Associations of substance use risk and protective factors and past-month drug use^a^ among a nationwide U.S. sample of Hispanic adolescents in 2014 and 2015 (*N* = 507).

	*M* or %	*R*	Odds ratio (95% *CI*)	
Risk and protective factors			Unadjusted Model	Adjusted Model
Ethnic pride	3.38	1.00–4.00	0.71 (0.45, 1.14)	0.63 (0.37, 1.06)
Acculturation	2.72	1.00–4.00	0.63 (0.32, 1.24)	0.54 (0.20, 1.44)
Traditionalism	2.28	0.00–3.00	1.45 (0.87, 2.43)	1.48 (0.80, 2.72)
Self-image	1.74	1.00–2.00	0.51⁎ (0.29, 0.92)	0.50⁎ (0.25, 0.99)
Stress level	2.53	1.00–4.00	0.49⁎⁎⁎ (0.35, 0.68)	0.56⁎⁎ (0.38, 0.84)
Coping	2.82	1.00–4.00	0.58⁎ (0.38, 0.89)	0.28⁎⁎⁎ (0.13, 0.57)
Peer use	47.8%	0.00–100	10.96⁎⁎⁎ (4.60, 26.13)	7.52⁎⁎⁎ (2.80, 20.22)
Self-control	3.01	1.00–4.00	0.58⁎⁎ (0.42, 0.82)	0.60⁎ (0.40, 0.91)
Goal setting	2.96	1.00–4.00	0.67⁎ (0.45, 0.98)	0.47⁎⁎ (0.27, 0.81)
Problem solving	2.78	1.00–4.00	0.57⁎ (0.370, 0.884)	0.40⁎⁎ (0.21, 0.77)
Self-efficacy	3.04	1.00–4.00	0.38⁎⁎ (0.19, 0.75)	0.35⁎⁎ (0.16, 0.78)
Media resistance	3.15	1.00–4.00	1.39 (0.77, 2.52)	1.18 (0.59, 2.36)
Intentions	10.4%	0.00–100	23.90⁎⁎⁎ (11.93, 47.90)	22.29⁎⁎⁎ (9.82, 50.58)

Note. *R* = range. *CI* = confidence interval. Adjusted models account for age, gender, race, living arrangement, parent education, and language spoken at home.

a Past-month drug use includes any reported use of alcohol, cigarettes, marijuana, inhalants, ecstasy, mushrooms, cocaine, methamphetamines, prescription pills, or heroin.

⁎ *p* < .05.

⁎⁎ *p* < .01.

⁎⁎⁎ *p* < .001.
